# Knowledge, attitude, and practices associated with rabies in villages with different dog vaccination statuses in Cambodia

**DOI:** 10.14202/vetworld.2021.2178-2186

**Published:** 2021-08-24

**Authors:** Bunsong Ung, Ketsarin Kamyingkird, Waraphon Phimpraphai

**Affiliations:** 1 Bio-veterinary Science Program, Faculty of Veterinary Medicine, Kasetsart University, Bangkok, Thailand; 2 Department of Animal Health and Veterinary Public Health, General Directorate of Animal Health and Production, Phnom Penh, Cambodia; 3 Department of Parasitology, Faculty of Veterinary Medicine, Kasetsart University, Bangkok, Thailand; 4 Department of Veterinary Public Health, Faculty of Veterinary Medicine, Kasetsart University, Bangkok, Thailand

**Keywords:** attitude, knowledge, practices, rabies

## Abstract

**Background and Aim:**

Rabies is a highly infectious but neglected zoonotic disease. Almost 99% of rabies-related human deaths are caused by dog-mediated rabies. Although canine rabies vaccination is highly effective and provides protection, nationwide rabies vaccination campaigns have been insufficient in Cambodia, resulting in a limited number of rabies vaccinated dogs. This study aimed to explore the rabies knowledge, attitude, and practices (KAP) among participants from both dog rabies vaccinated (DRV) and dog rabies unvaccinated (DRUV) villages located in the Kandal and Prey Veng Provinces, Cambodia.

**Materials and Methods:**

A cross-sectional survey was conducted with dog owners in Kandal and Prey Veng Provinces, Cambodia, during August and September 2020. The structural questionnaire collected general sociodemographic information and the KAP associated with rabies transmission, clinical signs, management, and control. The data were then analyzed using Wilcoxon rank-sum test and Chi-square statistics.

**Results:**

In total, 312 participants were interviewed: 137 participants from DRV villages and 175 from DRUV villages. Among them, 99.4% (310/312) had previously heard about rabies. Out of these 310, 93.5% (290/310) were aware that rabies is a fatal disease, while 96.5% (299/310) were willing to vaccinate their dog against rabies if the vaccination was provided for free. However, 32.9% (102/310) indicated that they would be willing to sell their own dog if it bit someone or showed aggression. More than one-third (115/310) of all the respondents had poor overall KAP regarding rabies. The respondents from DRV villages had significantly higher overall scores with regard to rabies KAP than those from DRUV villages (p<0.0001). According to the factors related to overall KAP, village type and education level were significantly associated with overall KAP of the respondents (p<0.0001).

**Conclusion:**

The rabies disease is recognized in Cambodia, and dog owners are willing to vaccinate their dogs if the vaccination is provided for free. The overall rabies-related KAP were poor among 30% of the respondents, and higher KAP scores were obtained for the DRV villages. The village type and education level were found to be associated with the different overall KAP of the participants.

## Introduction

Rabies is an acute encephalitic disease caused by the rabies virus. The disease affects virtually all mammals, and an infected species invariably die from the disease once the associated clinical signs have manifested [1,2]. Dog-mediated rabies contributes to 99% of rabies transmission to humans, and consequently, is the main cause of rabies-related human deaths [1,3]. The disease is estimated to cause 59,000 human deaths annually in over 150 countries, with 96% of cases occurring in Africa and Asia [3]. In Cambodia, a predictive model that was based on patients receiving rabies post-exposure prophylaxis (PEP) estimated that there were 810 rabies-related human deaths and at least 80,459 cases of dog bite injuries in 2007 [4]. Between 1998 and 2018, there were 87 encephalitis patients who died following a dog bite, with approximately 73% confirmed to be rabies positive [5]. Further, in Cambodia, around 22,000 patients received rabies PEP annually, and 90% of these cases involved dogs [5]. There are many major challenges related to rabies control in Cambodia, such as the lack of government funding for rabies activities and of national vaccination programs in the human and animal health sectors [5].

Although the transmission of the rabies virus primarily occurs through the saliva of dogs when they bite, scratch, or lick broken skin, there have been reports of rabies transmission from ingestion in an experimental setting [6]. Rabies-related human deaths caused by eating raw dog meat were reported in 84% (21/25) of non-bite exposures in the Philippines [7]. Worldwide, humans consume as many as 25 million dogs each year [8]. Cambodia has no animal welfare laws in place, including any national prohibition on the slaughtering of dogs for human consumption [9]. Further, there is evidence of dogs being traded and transported between provinces, with more than 3 million dogs estimated to be slaughtered for human consumption annually [10].

Mass rabies vaccination is a major integral component of rabies control. However, rabies elimination requires additional support components, including the effective involvement of the community and policy-makers, dog population evaluation and management, surveillance, and legislation [11]. Community support plays a crucial role in any rabies prevention and control program, so it is crucial to understand the community’s and dog owners’ knowledge, attitude, and practices (KAP) regarding rabies [12].

The study aimed to explore the rabies-related KAP among villagers residing in dog rabies vaccinated (DRV) and dog rabies unvaccinated (DRUV) villages in the Kandal and Prey Veng Provinces, Cambodia.

## Materials and Methods

### Ethical approval and informed consent

This study was approved by the Department of Animal Health and Veterinary Public Health, General Directorate of Animal Health and Production, Cambodia. Further, informed consent was obtained from all the participants.

### Study period and location

**Figure-1:**
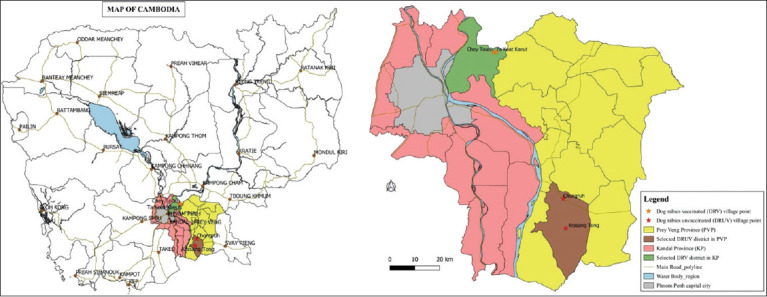
Map of study site [Source: Map prepared with the help of QGIS 3.12.3 Software].

The study was conducted from August to September 2020. The present study was carried out in the provinces of Kandal and Prey Veng (Figure-1). A total of four villages, namely, two villages from Kandal Province, where dogs had been vaccinated, and two villages from the Prey Veng Province, where they had not been vaccinated were selected based on their different dog rabies vaccination history. Through personal communication with district veterinarians, there were a total of 1913 dogs in the study area: 480, 530, 320, and 583 dogs in Chongruh, Krasang Tong, Chey Touch, and Ta Koat Kaeut villages, respectively. In the DRV villages, at least 80% of the total dog population had been vaccinated, and all the vaccines were supported by the World Organization for Animal Health (OIE). In addition, rabies vaccination and education campaigns had been conducted by the General Directorate of Animal Health and Production with financial support from the Institut Pasteur du Cambodge.

### Study design

A cross-sectional study design was used for the KAP survey of the participants. Face-to-face interviews were conducted using a standardized questionnaire designed by trained government district veterinarians. To test the validity and reliability of the questionnaire, a preliminary survey of 15 participants was conducted. Further, both qualitative and quantitative data were collected. A KAP questionnaire comprising four main parts with 27 questions was developed. The questionnaire covered the following topics:

·Sociodemographic and general information about the respondent, such as sex, age, education level, type of occupation, vaccination status of their dog, dog meat eating behavior, and whether they had heard of rabies before
·Rabies-related knowledge, such as causes, clinical signs, susceptible species, routes of transmission, effective ways to prevent rabies, and the most effective way to prevent canine rabies
·Attitudes toward rabies vaccination, such as willingness to vaccinate their dog against rabies, reasons for doing so, and human uptake of rabies PEP after being bitten by a vaccinated dog
·Practices related to rabies, including measures employed for the carcasses of suspected rabid dogs, dogs who had been bitten by a suspected rabid dog, dogs who bite people, and PEP attitudes after being bitten by a dog.

### Sample size

Two villages were selected for each of the canine rabies vaccination conditions (DRV and DRUV), and dog owners were then randomly selected from each group. The unit of study was the household level, and the KAP interview was conducted with one respondent from each household. Through personal communication with the chiefs of each village, there were a total of 1434 households in the study area, comprising 629 DRV and 805 DRUV households. The Taro Yamane’s formula with a margin error of 5% was used to obtain the required sample size of 312 respondents [13].


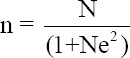


Where, n = Corrected sample size

N = Population size

e = Margin of error (MoE) (in proportion of one; if 5%, e = 0.05)

Substituting these values in the formula, we obtain the following equation:


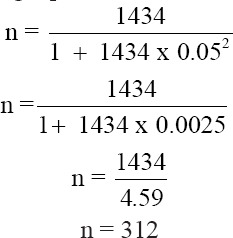


### Inclusion and exclusion criteria

In this survey, dog owners were randomly selected to be respondents, and only one family member from each household was surveyed. Participants need to be at least 18 years old to participate and be at home during the survey. The respondents who had never heard about rabies were excluded from the KAP sections of the survey due to the unreliable nature of any subsequent rabies-related data that would have been gathered from them.

### Statistical analysis

The data were collected and analyzed using MS Excel 2016 and R 3.6.1. The KAP scoring involved assigning a score of 1 for a correct answer and of 0 if the participant answered incorrectly or did not know the answer. There were 16 questions in the KAP sections, with a maximum possible point score of 32. These questions were divided as per the following categories: Knowledge (seven questions, 21 points); attitude (five questions, 5 points); and practices (four questions, 6 points). For the overall KAP rating, respondents who scored 16 or more points were deemed to have good KAP, and those with a score of below 16 were said to have a poor KAP. Descriptive statistical tests were performed to analyze all the important variables involved. The Wilcoxon rank-sum test was used to compare the rabies KAP, and overall KAP scores of participants employed in the two village types. The Chi-square/Fisher’s exact test was conducted to analyze the associated factors such as village type, sex, age, education level, occupation, and dog meat eating behavior with the overall KAP.

## Results

### Sociodemographic and general information about the respondents

A total of 312 participants were interviewed, of which 137 (43.9%) were from DRV villages and 175 (56.1%) were from DRUV villages. Almost two-thirds of the respondents in both groups were female, and a majority of the respondents were 36–55 years old. All participants were Buddhist, and primary school was the most common level of education. The most common occupation was that of a farmer, and all of the respondents were dog owners. Almost all the dogs owned by the respondents from the Kandal Province (DRV villages) had been vaccinated against rabies, whereas this was true for none of the vaccinated dogs owned by the respondents from the Prey Veng Province (DRUV villages). The vast majority of respondents had heard about rabies, and one-third of them had eaten dog meat (Table-1).

**Table-1:** Descriptive data of respondents.

Variable	DRUV villages, n (%)	DRV villages, n (%)	Total, n (%)
Sex (n=312)			
Female	90 (51.4)	91 (66.4)	181 (58)
Male	85 (48.6)	46 (33.6)	131 (42)
Age (n=312)			
18-35	49 (28)	21 (15.3)	70 (22.4)
36-55	98 (56)	79 (57.7)	177 (56.7)
56-70	28 (16)	37 (27)	65 (20.8)
Religion (n=312)			
Buddhism	175 (100)	137 (100)	312 (100)
Islam	0 (0)	0 (0)	0 (0)
Christianity	0 (0)	0 (0)	0 (0)
Educational level (n=312)			
Illiteracy	23 (13.1)	5 (3.6)	28 (9)
Primary	55 (31.4)	111 (81)	166 (53.2)
Secondary	42 (24)	19 (13.9)	61 (19.6)
High school	53 (30.3)	2 (1.5)	55 (17.6)
Vocational school	0 (0)	0 (0)	0 (0)
Tertiary	2 (1.1)	0 (0)	2 (0.6)
Type of occupation (n=312)			
Farmer	107 (61.1)	118 (86.1)	225 (72.1)
Labor worker	10 (5.7)	16 (11.7)	26 (8.3)
Housewife	11 (6.3)	1 (0.7)	12 (3.8)
Trader	31 (17.7)	0 (0)	31 (9.9)
Student	11 (6.3)	0 (0)	11 (3.5)
Other	5 (2.9)	2 (1.5)	7 (2.2)
Household with vaccinated dogs (n=312)			
Yes	0 (0)	133 (97.1)	133 (42.6)
No	175 (100)	4 (2.9)	179 (57.4)
Respondents who have eaten dog meat (n=312)			
Yes	58 (33.1)	41 (29.9)	99 (31.7)
No	117 (66.9)	96 (70.1)	213 (68.3)
Have heard about rabies (n=312)			
Yes	173 (98.9)	137 (100)	310 (99.4)
No	2 (1.1)	0 (0)	2 (0.6)
Know that rabies is a fatal disease (n=310)			
Yes	155 (89.6)	135 (98.5)	290 (93.5)
No	18 (10.4)	2 (1.5)	20 (6.5)
Know that rabies can be prevented by vaccination (n=310)			
Yes	169 (97.7)	137 (100)	306 (98.7)
No	4 (2.3)	0 (0)	4 (1.3)
Opinion regarding the effective way of preventing rabies (n=310)			
Human rabies vaccination	99 (57.2)	2 (1.5)	101 (32.6)
Dog rabies vaccination	74 (42.8)	135 (98.5)	209 (67.4)
Opinion regarding the most effective way of preventing rabies in dogs (n=310)			
Dog neutering	1 (0.6)	0 (0)	1 (0.3)
Dog rabies vaccination	167 (96.5)	136 (99.3)	303 (97.7)
Tying the dog at home	5 (2.9)	1 (0.7)	6 (1.9)
Knowledge of rabies clinical signs			
Behavior change (n=310)	8 (4.6)	47 (34.3)	55 (17.7)
Salivation (n=310)	158 (91.3)	136 (99.3)	294 (94.8)
Afraid of sunlight (n=310)	3 (1.7)	2 (1.5)	5 (1.6)
Attacking without provocation (n=310)	25 (14.5)	86 (62.8)	111 (35.8)
Runaway and dropped-down tail (n=310)	149 (86.1)	134 (97.8)	283 (91.3)
Depression (n=310)	4 (2.3)	4 (2.9)	8 (2.6)
Paralysis (n=310)	5 (2.9)	0 (0)	5 (1.6)
No idea (n=310)	3 (1.7)	1 (0.7)	4 (1.3)
Knowledge of the species that are susceptible to rabies			
Human (n=310)	167 (96.5)	137 (100)	304 (98.1)
Dog (n=310)	173 (100)	137 (100)	310 (100)
Cat (n=310)	45 (26)	63 (46)	108 (34.8)
Cattle (n=310)	22 (12.7)	11 (8)	33 (10.6)
Knowledge of routes of rabies transmission			
Dog bite/scratch (n=310)	169 (97.7)	137 (100)	306 (98.7)
Cat bite/scratch (n=310)	10 (5.8)	56 (40.9)	66 (21.3)
Infected saliva via wound (n=310)	5 (2.9)	108 (78.8)	113 (36.5)
Infected saliva via mucous membrane (n=310)	1 (0.6)	10 (7.3)	11 (3.5)
No idea (n=310)	3 (1.7)	0 (0)	3 (1)
Willing to get dog rabies vaccination if vaccination campaign is free of charge (n=310)			
Yes	162 (93.6)	137 (100)	299 (96.5)
No	11 (6.4)	0 (0)	11 (3.5)
Reasons for getting dog rabies vaccination if vaccination campaign is free of charge (n=299)			
Protect dog	49 (30.2)	2 (1.5)	51 (17.1)
Protect human	35 (21.6)	0 (0)	35 (11.7)
Protect dog and human	78 (48.1)	135 (98.5)	213 (71.2)
Willing to get dog rabies vaccination if vaccination costs 2 USD (n=310)			
Yes	141 (81.5)	122 (89.1)	263 (84.8)
No	32 (18.5)	15 (10.9)	47 (15.2)
Reasons for getting dog rabies vaccination if vaccination costs 2 USD (n=263)			
Protect dog	37 (26.2)	1 (0.8)	38 (14.4)
Protect human	30 (21.3)	0 (0)	30 (11.4)
Protect dog and human	74 (52.5)	121 (99.2)	195 (74.1)
Know that rabies PEP is needed after being bitten by a rabies vaccinated dog (n=310)			
Yes	165 (95.4)	136 (99.3)	301 (97.1)
No	8 (4.6)	1 (0.7)	9 (2.9)
Practices employed when dealing with a suspected rabid dog carcass (n=310)			
Burn	2 (1.2)	0 (0)	2 (0.6)
Bury	135 (78)	135 (98.5)	270 (87.1)
Throw away	2 (1.2)	0 (0)	2 (0.6)
Eat	22 (12.7)	2 (1.5)	24 (7.7)
Give to another	10 (5.8)	0 (0)	10 (3.2)
Sell	2 (1.2)	0 (0)	2 (0.6)
Practices for a dog that bites (n=310)			
Do nothing	33 (19.1)	2 (1.5)	35 (11.3)
Confine for 10 days	1 (0.6)	32 (23.4)	33 (10.6)
Kill and send the head for testing	0 (0)	4 (2.9)	4 (1.3)
Kill and eat the dog	34 (19.7)	0 (0)	34 (11)
Sell the dog	29 (16.8)	73 (53.3)	102 (32.9)
Give the dog to another	65 (37.6)	26 (19)	91 (29.4)
Kill and bury	10 (5.8)	0 (0)	10 (3.2)
Other	1 (0.6)	0 (0)	1 (0.3)
Practices for a dog bitten dog by a suspected rabid dog (n=310)			
Do nothing	4 (2.3)	0 (0)	4 (1.3)
Treat the wound only	2 (1.2)	0 (0)	2 (0.6)
Kill the dog	32 (18.5)	1 (0.7)	33 (10.6)
Sell the dog	38 (22)	58 (42.3)	96 (31)
Give dog to other	79 (45.7)	57 (41.6)	136 (43.9)
Bring the dog to see VAHW	13 (7.5)	2 (1.5)	15 (4.8)
Confine for less than two weeks	5 (2.9)	19 (13.9)	24 (7.7)
Practices employed if bitten by a dog			
Clean the wound with running water and soap (n=310)	4 (2.3)	123 (89.8)	127 (41)
Use antiseptic to clean the wound (n=310)	0 (0)	33 (24.1)	33 (10.6)
Rabies PEP (n=310)	147 (85)	130 (94.9)	277 (89.4)
Traditional treatment (n=310)	3 (1.7)	4 (2.9)	7 (2.3)
Do nothing (n=310)	2 (1.2)	0 (0)	2 (0.6)

DRV=Dog rabies vaccinated, DRUV=Dog rabies unvaccinated, PEP=Post-exposure prophylaxis, VAHW=Village Animal Health Worker

### Knowledge of rabies

Of the 312 respondents, 310 completed the KAP sections of their questionnaire, and more than 90% of them recognized rabies as a fatal disease that is preventable through vaccination. Although dog vaccination was noted as an effective way of preventing rabies, about one-third (101/310) of the respondents believed human rabies vaccination to be better for rabies prevention. Salivation (294/310), runaway dogs with their tails hanging straight down (283/310), and attacking without provocation (111/310) were most frequently identified as clinical signs of rabies. Further, changes in behavior, a fear of sunlight, depression, and paralysis were infrequently identified. Almost all the respondents knew that humans (304/310) and dogs (310/310) were susceptible to rabies, and less than 50% mentioned cats (108/310) or cattle (33/310) (Table-1). However, none of the respondents knew that wild animals such as bats, rats, and monkeys are also susceptible species to rabies. A dog bite or scratch was mentioned as one of the major routes of rabies transmission by most respondents (306/310); but in the DRV villages, a cat bite or scratch (56/137) and infection by saliva on a wound (108/137) were also mentioned by a higher number of respondents (Table-1). The median score for rabies knowledge in the DRV and DRUV villages was 11 (lower quartile=11 and upper quartile=13) and 9 (lower quartile=8 and upper quartile=9), respectively. There was a statistically significant difference in the rabies knowledge scores between the two village categories (p<0.0001) (Table-2).

**Table-2:** Overall score of respondents between rabies vaccinated and unvaccinated villages (n=310).

Variable	Lower quartile	Median	Upper quartile	p-value
Overall scores of rabies knowledge (21 points)				
Rabies vaccinated villages	11	11	13	<0.0001
Rabies unvaccinated villages	8	9	9	
Overall scores of attitudes toward rabies (5 points)				
Rabies vaccinated villages	5	5	5	<0.0001
Rabies unvaccinated villages	3	3	5	
Overall scores of rabies practices (6 points)				
Rabies vaccinated villages	3	3	4	<0.0001
Rabies unvaccinated villages	2	2	2	
Overall scores of KAP (32 points)				
Rabies vaccinated villages	18	20	21	<0.0001
Rabies unvaccinated villages	13	14	16	

KAP=Knowledge, attitude, and practices

### Attitudes toward rabies

A majority of the respondents were willing to vaccinate their dog against rabies if the vaccination was provided for free (96.5%) or if they had to pay 2 USD (84.8%) for it. The reason provided for this by most of the respondents was that it would protect human and canine lives (71.2% free and 74.1% paid for). Almost all respondents (97.1%) were aware that the rabies PEP vaccination is needed even if the dog that bit them had been vaccinated for rabies (Table-1). The median score of the attitudes toward rabies in the DRV villages was 5 (lower and upper quartiles=5) and that in the DRUV villages was 3 (lower quartile=3 and upper quartile=5). There was a statistically significant difference between attitudes toward rabies scores when comparing the two village conditions (p<0.0001) (Table-2).

### Rabies practices

More than three-fourths of the respondents (87.1%) said that they would bury the carcass themselves if they had a suspected rabid dog that died, and 8.4% said that they would eat or sell the carcass. If their dog bit someone, 62.3% of the dog owners would sell or give their dog to someone else. However, 23.4% of the respondents from the DRV villages did report that they would confine their dog for 10 days to observe it for the clinical signs of rabies if it bit someone. Three-fourths of dog owners (74.8%) said that they would give away or sell their dogs if the dog was bitten by a suspected rabid dog, whereas 10.6% would kill the dog, and 4.8% would take the dog to a Village Animal Health Worker. The post-bite care was reported most frequently in the DRV villages involved rabies PEP and cleaning the wound with running water and soap. However, in DRUV villages, while 85% of the participants reported that they would receive rabies PEP following a dog bite, only 2.3% mentioned that they would wash the wound with soap and running water. Further, in both DRV and DRUV villages, 2.9% of the total respondents would apply traditional treatments or do nothing after a dog bite (Table-1). The median rabies practice score for the DRV and DRUV villages was 3 (lower quartile=3 and upper quartile=4) and 2 (lower and upper quartiles=2), respectively. There was a statistically significant difference between the rabies practice scores of the two village categories (p<0.0001) (Table-2).

### Association of factors with rabies KAP and overall KAP scores

The respondents from the DRV villages had higher separate KAP scores as well as overall KAP scores than the DRUV villages (Table-2). Independent of whether a respondent was from the DRV or DRUV villages, education level had a significant association with overall KAP (p<0.0001). More than half of the respondents, 62.9% (195/310) had a good overall KAP. The analysis of the associations between each of the outcome variables and the participants is presented in Table-3.

**Table-3:** Factors associated with overall KAP (n=310).

Factors	Good overall KAP (16-32), n=195	Poor overall KAP (0-15), n=115	p-value χ^2^/Fisher’s exact test
Type of villages			
DRV villages	133	4	<0.0001
DRUV villages	62	111	
Sex			
Female	118	61	0.2432
Male	77	54	
Age			
18-35	42	26	0.9757
36-55	112	65	
56-70	41	24	
Education level			
Illiteracy	10	18	<0.0001
Primary	123	41	
Secondary	33	28	
High school and tertiary	29	28	
Type of occupation			
Farmer	142	82	0.8611
Labor worker	17	9	
Housewife	7	5	
Trader	16	14	
Student	8	3	
Other	5	2	
Dog meat consumption			
Yes	61	37	0.9707
No	134	78	

KAP=Knowledge, attitude, and practices, DRV=Dog rabies vaccinated, DRUV=Dog rabies unvaccinated

## Discussion

The present study was undertaken to assess the rabies KAP of the members of the rural communities residing in the Kandal and Prey Veng Provinces to better understand the challenges associated with rabies control in rural Cambodia. Before this work, there was a study that identified the knowledge of rabies and dog-related behavior in rural Siem Reap, Cambodia, during December 2013 and January 2014 [14]. As per our knowledge, this is the first study to demonstrate the KAP of village residents where there was a difference in the canine rabies vaccination status between villages.

We found that all of the 175 respondents from the DRUV villages did not vaccinate their dogs against rabies. This finding was higher than the previous findings from Ethiopia [15,16], Cambodia [14], and India [17]. The study found that 99.4% of the respondents had heard about rabies, which was higher than previous findings from Ethiopia (77.9%) [15], Cambodia (86.9%) [14], Pakistan (89.4%) [18], and Bhutan (98%) [19]. This study not only highlights the need to enhance dog rabies vaccination campaigns but also reveals a high level of awareness of rabies within this endemic country.

This study found that a high proportion of respondents (93.5%) were aware that rabies is a fatal disease. This statistic was higher than that found in Bangladesh (79.8%) [20], Pakistan (72.4%) [21], Cambodia (71.7%) [14], and Bhutan (93%) [19]. The study also found that a majority of the respondents (98.7%) knew that rabies is prevented by vaccination, which was higher than the corresponding findings for Bangladesh (78.3%) [20], Pakistan (69.8%) [21], and Ethiopia (65.9%) [15]. More than two-thirds of the respondents in our study (67.4%) said that dog rabies vaccination is an effective way of preventing rabies. This finding was lower than a prior finding from Pakistan, which reported 77.6% [18]. The proportion of the respondents who knew that rabies is transmitted through dog bites (98.7%) was higher in this survey than in the previous studies from Cambodia (98.6%) [14], Pakistan (62.9%) [18], and India (7.06%) [22], although it was lower in a study conducted in Bhutan (99%) [19]. This indicates that people are aware that rabies is a highly fatal disease and that dogs can pass rabies onto humans.

In this study, the number of respondents who were willing to vaccinate their dogs if vaccination was provided for free (96.5%) was higher than those in Ethiopia (69.8%) [15]. Interestingly, more than 84% of the respondents said that they were willing to pay 2 USD for a dog rabies vaccination. This was different from the study from Pakistan, in which only 57.9% of the participants reported being able and willing to afford human rabies vaccination [21]. This positive attitude toward canine rabies vaccination is a good indicator for the potential success of future dog rabies vaccination campaigns in Cambodia.

In this study, <8% of the respondents claimed that they would eat the carcass of a suspected rabid dog. This was starkly different from a previous study conducted in Cambodia, which found that 28% of the respondents ate animals that had been found dead [23]. A majority of the respondents (62.3%) said that they would sell or give away a dog who bites or is aggressive. These practices go against the scientific recommendation that a dog who bites should be confined for 10 days to observed for the clinical signs of rabies following a bite incident [3]. In addition, if a dog was bitten by a suspected rabid dog, around two-thirds of the respondents (67.6%) from the DRUV villages said that they would sell or give away the dog. These practices also go against the CDC recommendation that dogs that have never had a rabies vaccine and have been exposed to a rabid dog should be euthanized [24]. These findings indicate a huge gap between dog owner practices and the scientific recommendations for rabies control and prevention.

A critical finding in this survey was that the respondents from the DRV villages scored higher with regard to rabies KAP and overall KAP than those from the DRUV villages. The education level of the participants was significantly associated with the overall KAP of rabies, and illiterate respondents presented the lowest overall KAP. The poor KAP found among the illiterate respondents from the DRV and DRUV villages in both studied provinces are similar to the previous survey carried out in DRUV villages in the Siem Reap Province, Cambodia [14], Ethiopia [25], and Brazil [26]. This is probably linked to the fact that education can help people gain more knowledge regarding diseases. This is also corroborated by our finding that there was an enormous gap in terms of rabies awareness between the respondents from the DRV and DRUV villages, which might be due to the rabies education implementation during dog rabies vaccination campaigns.

One limitation of this study is that only four villages (two DRV and two DRUV) were studied. However, extensive surveys in other villages in both urban and rural provinces of Cambodia are required to gather comprehensive KAP data that can provide a baseline for future disease control.

## Conclusion

Rabies is recognized in Cambodia, and dog owners are willing to vaccinate their dogs if the vaccination is provided free of charge. The overall rabies KAP were poor among 30% of the respondents, and higher KAP scores were obtained for the DRV villages. It was found that village type and education level were associated with the different overall KAP employed by the participants.

## Recommendations

It is crucial to implement canine rabies vaccination and education campaigns in other villages to manage and control the disease effectively in the entire country. Furthermore, illiterate groups should be given priority in rabies education campaigns. To contribute to the global strategic plan of having zero human deaths from dog-mediated rabies by 2030, strengthening of dog rabies surveillance systems is also vital.

## Authors’ Contributions

BU and WP: Planned the entire research work, carried out the data entry, analysis, and interpretation. BU, WP, and KK: Contributed the same when training government district veterinarians about data collection and writing the manuscript. All the authors have read and approved the final manuscript.
